# Ethical Tenets of PRN Medicines Management in Healthcare Settings: A Clinical Perspective

**DOI:** 10.3390/pharmacy9040174

**Published:** 2021-10-22

**Authors:** Mojtaba Vaismoradi, Cathrine Fredriksen Moe, Flores Vizcaya-Moreno, Piret Paal

**Affiliations:** 1 Faculty of Nursing and Health Sciences, Nord University, 8049 Bodø, Norway; cathrine.f.moe@nord.no; 2 Department of Nursing, Faculty of Health Sciences, University of Alicante, 03080 Alicante, Spain; flores.vizcaya@ua.es; 3 WHO Collaborating Centre, Institute of Nursing Science and Practice, Paracelsus Medical University, 5020 Salzburg, Austria; piret.paal@pmu.ac.at

**Keywords:** ethics, nurse, medication safety, medicines management, patient safety, *pro re nata*, PRN

## Abstract

Prescription and administration of *pro re nata* (PRN) medications has remained a poorly discussed area of the international literature regarding ethical tenets influencing this type of medication practice. In this commentary, ethical tenets of PRN medicines management from the clinical perspective based on available international literature and published research have been discussed. Three categories were developed by the authors for summarising review findings as follows: ‘benefiting the patient’, ‘making well-informed decision’, and ‘follow up assessment’ as pre-intervention, through-intervention, and post-intervention aspects, respectively. PRN medicines management is mainly intertwined with the ethical tenets of beneficence, nonmaleficence, dignity, autonomy, justice, informed consent, and error disclosure. It is a dynamic process and needs close collaboration between healthcare professionals especially nurses and patients to prevent unethical practice.

## 1. Introduction

Medicines management is a complex process and has a multidisciplinary identity indicating the need for close collaboration between healthcare professionals including physicians, pharmacists, nurses, and patients [1]. The main tool for an effective collaboration is interaction between healthcare providers that can reduce adverse drug events given the significance of communication lines in the prevention of medications errors [2]. 

Ambiguous orders, incorrect interpretation of orders, and inappropriate monitoring of medications reflect insufficient and ineffective multidisciplinary collaboration between healthcare professionals involved in the medication process that perpetuates medication errors [3,4]. 

The multidisciplinary healthcare team can optimise medicines management in terms of the reduction of polypharmacy, improvement of adherence to medications, and balancing risks and benefits of medications [5]. Systematic assessment and monitoring of the medication process and related side effects and adverse drug reaction (ADRs) can minimise the possibility of errors [6]. The collaborative approach has great potential to improve medication safety if it is cohesive and is practiced based on clearly designed roles and responsibilities [7]. The best outcome of the medication process is achieved when pharmacists, physicians and nurses undertake their assigned roles and collaborate to ensure clinical medication safety [8,9].

## 2. PRN Medicines Management

*Pro re nata* (PRN) has been defined as the administration of medications by the nurse based on the patient’s immediate needs for medications rather than at routine and predetermined times [10,11]. The physician prescribes the medication, and the nurse makes a decision on its administration based on the patient’s request to receive medications. The nurse’s decision-making is based on creating a mutual understating and feeling of responsibility between the patient and the nurse [11,12,13]. Additionally, the nurse has the great responsibility of documenting the medication process and reporting adverse events, near misses and errors to pharmacists [14] who have the required authority to withhold medications in the best interest of the patient and perform related follow up discussions with the physician [15,16].

Common medications used as PRN are psychotropic, psycholeptic, antipsychotics, neuroleptics, anxiolytics, sedatives, hypnotics, and analgesics [11,12,13,17,18]. Our knowledge of errors associated with PRN medications is very limited. However, not acting on PRN medication requests by the patient within 15 min has been defined as the medication error [19]. Additionally, 9–40% of medication errors in intensive care units have been attributed to PRN medications [20] and, in general wards, 23% of PRN medications are accompanied with an unclear indication for prescription and administration. Moreover, 36% of PRN medications are not stopped though they are not administered at all during hospitalisation [21]. 

PRN medicines management provides a flexible care condition for the patient to submit the medication request to the nurse with the aim of relieving his/her physical and psychological suffering who has the legal and ethical responsibility to decide on the appropriateness of pharmacological interventions based on the physician order [22,23]. There is no strong evidence from randomised clinical trials to support the process of PRN medications’ prescription and administration. Therefore, it is often practised based on clinical experience and work routines [24]. 

It is also influenced by ethical tenets, law, healthcare policies, institutional guidelines, patients’ and healthcare professionals’ values and beliefs [17,18]. Moreover, socio-demographic characteristics of the patient as gender, ethnicity, and education level can influence PRN medication practice. For instance, old age, female gender, being black, living in a one-person household, and poor health literacy can be more associated with the use of PRN medications [25,26]. 

Nevertheless, there is no integrated and comprehensive knowledge about which ethical tenets influence PRN medications. Therefore, the aim of this commentary was to discuss ethical tenets influencing PRN medicines management from the clinical perspective. 

## 3. Ethics and PRN Medicines Management 

**Figure 1:**
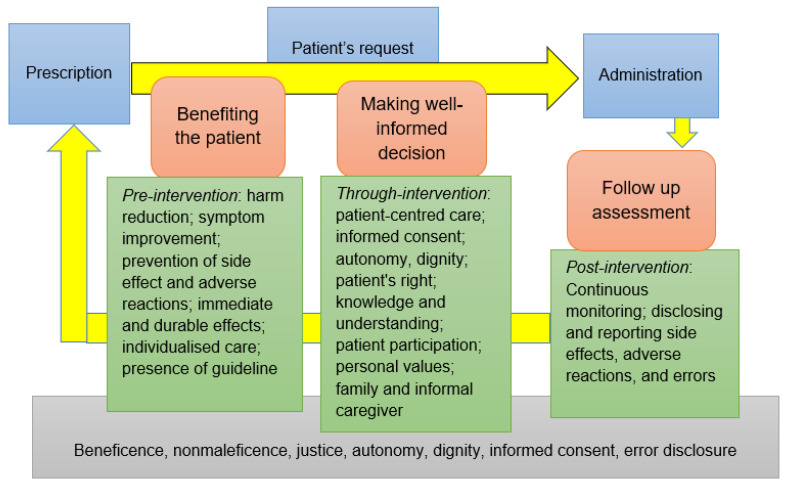
PRN medicines management and ethics from the clinical perspective.

Our review findings regarding ethical tenets influencing PRN medicines management from the clinical perspective have been summarised using the following author-made categories: ‘benefiting the patient’, ‘making well-informed decision’, and ‘follow up assessment’ as pre-intervention, through-intervention, and post-intervention aspects, respectively (Figure 1). Each category describes the clinical process of PRN medicines management in connection to ethics and discusses how unethical practice can be avoided.

### 3.1. Ethical Tenets of Benefiting the Patient

#### 3.1.1. Beneficence and Nonmaleficence

PRN medicines management starts with the patient’s verbal request for medications aiming at relieving his/her physical and/or mental suffering. The observation of the patient’s clinical sign and behavioural clues also can help the nurse identify his/her need for PRN medications [27]. 

As the pre-intervention aspect of PRN medication practice, the nurse should consider the ethical tenets of beneficence and nonmaleficence indicating his/her professional obligation to do no harm. The nurse should refrain from exposing the patient to any health-related negative consequences of medications and take all precautionary measures to meet the patient’s need, but not to add to his/her suffering, through selecting the most suitable medications [28,29].

Despite the benefits of PRN medications, their inappropriate and unnecessary prescription and administration can be associated with polypharmacy (≥5 medicines), overdosing, over- or under-use, and the patient’s disagreement given administration without the full disclosure of information about medications to the patient [30,31]. The nurse should play the role of the patient’s advocate and prioritise the benefit to the patient through the assessment of medication effectiveness, probability of harm, side effects and adverse reactions, and their impact on patient’s wellbeing and health, before making a decision on medication administration. 

The use of PRN medications should be both evidenced-based and patient-oriented to have ethical support and legitimacy for application in practice. The short-term effect of PRN medications and their long-term harm should be balanced. The use of PRN medications should not only be grounded in the empirical evidence of treatment efficacy, but also should be grounded in personal values to enable taking moral decisions. Instrumental rationality as the pursuit of any means for achieving desirable results is the dominant mode of thought in the current time that focuses mostly on the aim and outcome of healthcare interventions. However, to assess whether to prescribe and administer PRN medications, ethical tenets state that the nurse should go beyond instrumental rationality and consider the full range of humanistic possibilities [32], with the consideration of utmost pragmatic benefit of the medication to the patient in decision-making situations [33]. Nurses should assess the patient’s clinical status and ensure that his/her request for PRN medications reasonably and safely can meet his/her needs and does not lead to medication abuse [34], especially for mentally ill and cognitively challenged patients that may have a higher chance of self-harm by medications [22].

#### 3.1.2. Justice 

The ethical tenet of justice preserves the equal right to access to medication therapy in a similar manner to all patients regardless of their age, race, ethnicity, gender, and ability to pay [34,35]. Unconscious or implicit bias such as stigmatising patients can damage the healthcare provider–patient relationship and consequently the caring process in terms of making inappropriate clinical decisions leading to healthcare disparities and different outcomes. Healthcare providers should seek patients’ perspectives and prevent situations in which stereotypical and negative responses may be given to the patient’s requests [36,37,38]. Nevertheless, the nurse needs to make decisions on PRN medication administration based on the physiological and psychological characteristics of each patient in order to provide individualised care [25,26]. This ethical tenet supports the need for balancing general evidence-based practice and the selective application of such evidence in the clinical context of individualised care [39]. Therefore, it encompasses demographic and health-related characteristics, perceived and expressed preferences of the patient, nurse–patient relationships, and care philosophy within the workplace [39].

PRN medicines management requires the close engagement and collaboration between the patient and the nurse. Although some patients are willing to take more responsibility for their own care, some other patients prefer healthcare professionals to be more attentive and proactive [40]. 

The patient’s perspective of the risk and benefit of medications and cost-effectiveness, given the patient’s level of understanding and health literacy, should be sought [41]. Both the patient and the nurse should reach the common understanding that every increase in the number of PRN medications administered during the medication round can increase the risk of medication errors by 15%, along with the increased possibility of adverse drug events and reactions due to polypharmacy [12,42,43]. 

The nurse should also resolve the ethical concerns of the use of various medications with different effectiveness levels and help with the selection of the most effective medication with the least harmful effect [44]. A practical strategy can be the use of medication guidelines that help prevent medication errors, reduce side effects and adverse reactions given their concentration on systemic risk reduction and potential benefits for all patients [45,46]. However, there is a lack of research-based PRN medicines management guidelines and the available suggested ones in the international literature have been developed based on work routines and traditions of medication practice specialised to healthcare settings with a low possibility of generalisation to other healthcare settings [11]. Until an appropriate PRN medication guideline is developed and tested, the STOPP/START criteria for the medication process can also be taken into account for screening the possibility of abuse and preventing harm [30]. Additionally, noncompliance to PRN medications given their impact on the overall effectiveness of the medication process should be monitored [47]. 

### 3.2. Ethical Tenets of Making Well-Informed Decision

#### 3.2.1. Autonomy and Dignity

As the through-intervention aspect, attention should be paid to the patient’s autonomy and dignity in order to make the appropriate decision by the nurse on the administration of PRN medications. 

The nurse has an ethical responsibility to respect the patient’s right for receiving information about PRN medications in terms of the type of medication, medication’s side effects, voluntary notion of taking the medication, and freedom to accept or refuse it based on the given information. 

Care is a dynamic process and clinical practice moves along the continuum between ‘autonomy’ and ‘paternalism’, as well as between ethically reflective and non-reflective practice [48]. Nurses often find it difficult to practice paternalistic and ignore the patient’s autonomy, even if their professional knowledge is contrary to the patient’s preference and perspective of what is the best for him/her. 

Lack of attention to and inconsideration of the patient’s perspectives when deciding on the process of healthcare is missed-nursing care and is interpreted by the patient as an unmet care need [49]. A main part of advocacy is to prioritise the patient’s healthcare needs and remain committed to meeting them based on his/her preferences [50]. 

The patient’s autonomy is the cornerstone of moral care [51] encompassing the patient’s right to choose the suitable type of care, which creates an obligation in healthcare professionals to respect the patient’s choice leading to the feeling of dignity [28]. 

#### 3.2.2. Informed Consent

Informed consent as an ethical panacea counters autocratic and paternalistic healthcare practice and emphasises the patient’s right to be fully informed and to be able to freely choose between available therapeutic modalities [52]. The patient’s lack of trust in medications with regard to their effectiveness is a barrier to fully comply with PRN medicines management [53]. The patient should be informed of the benefits of PRN medications in terms of the improvement of physical and psychological symptoms and overall wellbeing. The patient needs clear information and support in order to make a decision with full consent based on accurate, complete, and unbiased information about medications. It should be delivered in a way that he/she can understand and act upon [54,55,56]. This approach enhances the patient’s self-agency, and motivation for involvement in the recovery process [57]. 

The patient should be empowered to choose something that aligns with their own perspective of life and moral values [54]. It is not uncommon that the patient refuses to receive PRN medications for pain management, because of his/her personal beliefs and values or having concerns about risks associated with medications [58]. The most common barrier to patient participation in the medication process is his/her level of understanding of medications and their positive effects [59], and the common practice should be to inform the patient and his/her informal caregiver of the most common and serious medication’s side effects. On the other hand, not informing the patient of all types of medication side-effects especially rare and non-serious ones to empower the patient to decide on taking or not taking medications can undermine respect for the patient’s autonomy [60]. 

The patient’s participation in decision-making for PRN medications highly depends on his/her mental capacity to understand information and decide upon it [17]. Sometimes PRN medications including sedatives are prescribed to the patient who refuses care and may harm himself/herself, but the medication can improve the patient’s collaboration with care [61]. The cases of the involuntary PRN medication administration of psychotropics, hypnotics or sedatives for older people with cognitive diseases or patients with mental disabilities [62,63] requires the interpretation of their symptoms and behaviours by the nurse, but it can cause concerns, moral uncertainty and distress, especially when the nurse takes the paternalistic role and coerces the patient who resists receiving medications [51,64]. In such cases, open discussions with informal caregivers and families of the patient about dangers posed by non-adherence to PRN medications [65,66] without invalidating the user response to the medication suggestion is an ethical intervention and leads to active involvement in medication self-management [67,68]. 

### 3.3. Ethical Tenets of Follow up Assessment

#### Error Disclosure

As the post-intervention aspect, the nurse’s ethical responsibility encompasses the continuous monitoring and follow up of the consequences of PRN medications, detection of errors, and assessment of their impacts on healthcare outcomes.

Controversies surrounding errors after the administration of PRN medications by the nurse encompass the insufficient assessment of medication outcomes, lack of monitoring side effects, and inappropriate documentation with regard to the medication’s indication and doses [20,69]. 

The patient is partner in PRN medicines management and can be involved in recognising and reporting symptom improvements, medications’ side effects and adverse reactions. Additionally, he/she should be motivated, informed and educated on how to report them, and how their reports lead to the improvement of the medication process [70,71], wellbeing and healthcare outcomes [72,73]. 

Disclosing the consequences of the medication process with the patient and requesting reporting and feedback on the patient’s side demonstrates the healthcare provider’s respect for the patient’s dignity and involvement in decision making. 

Medication errors generally damage the patient’s trust in healthcare professionals [74] and the nurse has the ethical duty to acknowledge mistakes and voluntarily disclose them to the patient and family members and expect fair reactions during the disclosure process [75]. 

Ethical obligations, professional guidelines, and patient safety principles all support the prompt disclosure of harmful medical errors [76]. Reporting and disclosing errors are prevailed by the ethos of silence, secrecy, and shame [77] and are often impeded by the perspective from which the patient’s harm is not apparent, and the error can be ignored and hidden. However, it can have negative implications for patient safety culture and creates changes in harmful medication routines [78,79,80]. Additionally, it serves nonmaleficence and beneficence to the extent that it prevents further harm to the patient or to other patients who may request the same medication [81] and can preserve and restore the patient’s feeling of dignity and respect [82,83]. Deprescribing as the process of withdrawal of inappropriate medications also can prevent the patient’s exposure to probably inappropriate medications [43,84]. 

## 4. Safeguarding PRN Medicines Management in Connection to Ethical Care

The third WHO Global Patient Safety Challenge: Medication Without Harm proposes solutions to safeguard medication practice and reduce all types of medication harm by 50% in the next 5 years across the globe [85]. Accordingly, healthcare systems have been obliged to employ all their capacities to reach the goal of a safer medication process and avoid, prevent, or correct adverse drug events during prescribing, order communication, compounding, distribution, administration, education, follow up, and monitoring of medications [86]. It also includes safe administration of PRN medications by the nurse, which can directly improve safety of the healthcare system. The prevalence of potentially inappropriate medications is 5–94% with an incidence of preventable adverse medication events of 15/1000 person-years [65]. In Europe, the rate of medication errors has been estimated to be 0.3–9.1% at prescription and 1.6–2.1% at administration stages [87]. Medication errors can lead to patients’ disability and death, and an estimated healthcare cost of USD 42 billion annually [88]. Therefore, policies have been articulated by international healthcare organisations to enhance attention by healthcare systems to the problem of medication safety and strengthening science-based systems for improving safe medication practice [89,90,91]. 

Discussion regarding the ethical tenets of PRN medicines management can shape the fundamental principles of law, indicating what is permissible to practice legally and what must be done to ensure the safety of medicines management [28]. For instance, the duty to use knowledge and skills by healthcare staff and prevent any failure leading to patient harm has its root in ethics and the breach of this duty is considered negligence leading to legal consequences [92,93,94].

The use of health information technology has been shown to be promising in the improvement of the safety of the medication process [74]. For instance, online communication platforms can help with interprofessional interactions through structuralising medication reviews and making appropriate decisions on the prescription and administration of PRN medications without delay based on the patient’s request [95]. However, the development of the best practice model leading to an ethical medication process and less patient harm requires further research [13,96]. 

The nurse as pharmacovigilant intermediary agent in medicines management is responsible for the prevention and detection of adverse drug events and errors [97]. The role model and active-learning strategies can be used for articulating and internalising ethical values by the nurse and improving the nurse’s competencies regarding how to apply the ethical, legal, and social principles of medication safety in clinical practice [98,99,100]. 

## 5. Limitations

The gap of knowledge in the international literature regarding PRN medicines management and the insufficient number of empirical studies on the ethical considerations of PRN medications hindered the researchers in conducting a systematic review. However, the researchers performed a comprehensive and broad search in various general and specialised databases without time restrictions using different search phrases to ensure of the inclusion of studies on PRN and ethics in this narrative review. It should be noted that the selected articles were conducted in hospitals and long-term healthcare settings indicating heterogeneity of their focus and findings. Therefore, this might have influenced integrating their findings into our review. 

## 6. Conclusions

PRN medicines management is a dynamic process with the involvement of all healthcare professionals and the close collaboration between the patient and the nurse, which is mainly intertwined with the ethical tenets of beneficence, nonmaleficence, autonomy, dignity, justice, informed consent, and error disclosure. The improvement of PRN medicines management based on these tenets requires education and training, as well as the improvement of nurses’ attitudes. 

The administration of PRN medications by the nurse with the consideration of ethical tenets requires:

- Prevention of harm and abuse;
- Selection of effective medications with the least side effect and adverse reactions;
- Creation of balance between the short- and long-term effects of medications;
- Consideration of the patient’s perspectives and personal values;
- Provision of information and education to the patient and family members;
- Development of PRN guidelines;
- Teamwork and multidisciplinary collaboration;
- Participation of the patient and family caregivers;
- Continuous monitoring and follow up;
- Disclosure and reporting of medication errors, side effects and adverse reactions.

Empirical studies should be conducted to explore nurses’ perspectives and experiences of the ethical considerations of PRN medicines management in healthcare settings.
